# Relationship between HLA-DRB1 shared epitope alleles and peripheral blood monocyte counts in Japanese patients with rheumatoid arthritis

**DOI:** 10.1371/journal.pone.0336214

**Published:** 2025-11-05

**Authors:** Daisuke Hiraoka, Jun Ishizaki, Kenta Horie, Kensuke Oryoji, Shin-ichi Mizuki, Katsuto Takenaka

**Affiliations:** 1 Department of Hematology, Clinical Immunology and Infectious Diseases, Ehime University Graduate School of Medicine, Toon, Ehime, Japan; 2 The Center for Rheumatic Diseases, Japanese Red Cross Matsuyama Hospital, Matsuyama, Ehime, Japan; 3 Ropponmatsu Rheumatology and Immunology Clinic, Fukuoka, Japan; Fondazione Don Carlo Gnocchi, ITALY

## Abstract

**Objectives:**

To investigate the relationship between Human Leukocyte Antigen-DR beta 1 (HLA-DRB1) shared epitope (SE) alleles and peripheral blood monocyte counts in disease-modifying antirheumatic drug-naïve patients with rheumatoid arthritis (RA), and also the relationship between specific SE alleles and anti-cyclic citrullinated peptide antibody (anti-CCP Ab) titers.

**Methods:**

This retrospective single-center study included 86 Japanese patients with RA. HLA-DRB1 genotyping was performed, and SE alleles associated with a high risk of developing RA were classified into the S_2_ (*04:01) and S_3P_ (*01:01, *01:02, *04:04, *04:05, *04:08, and *10:01) categories. Patients were stratified based on monocyte count tertiles. The relationships between monocyte counts at diagnosis and clinical, serological, and genetic factors were analyzed. Logistic regression was used to identify independent factors associated with high monocyte counts.

**Results:**

SE-positive patients, particularly those with S_3P_ alleles, had significantly higher monocyte counts than SE-negative patients. A multivariate analysis revealed that male sex and S_3P_ positivity, particularly HLA-DRB1 *01:01 or *04:05, were independently associated with higher monocyte counts. Patients carrying at least one S_3P_ allele had significantly higher anti-CCP Ab titers, with patients homozygous for HLA-DRB1 *04:05 having the highest levels. A similar relationship was not found with HLA-DRB1 *01:01 despite its strong effect on monocyte counts.

**Conclusions:**

This is the first study to demonstrate a significant association between SE alleles and peripheral blood monocyte counts in RA. The results obtained suggest that specific SE alleles, particularly S_3P_ alleles, contribute to the early pathogenesis of RA by enhancing monocyte-driven immune activation and anti-CCP Ab production.

## Introduction

Rheumatoid arthritis (RA) is a systemic autoimmune disease characterized by chronic synovial inflammation, joint destruction, and the production of autoantibodies, such as rheumatoid factor (RF) and anti-citrullinated protein antibodies (ACPAs) [1]. Genetic and environmental factors are both involved in its etiology. Although several genetic factors have been reported, the shared epitope (SE) was the first to be identified and remains the genetic factor most strongly associated with the development of RA [2]. SE refers to Human Leukocyte Antigen-DR beta 1 (HLA-DRB1) alleles that contain the amino acid sequences QKRAA, QRRAA, or RRRAA at positions 70–74 of the molecule [3,4]. SE enhances the binding affinity of HLA-DRB1 to citrullinated proteins, leading to the production of ACPAs through antigen presentation [4]. Importantly, not all SE alleles confer equal risk. According to refined classifications proposed by Tezenas et al., S_2_ alleles (e.g., *04:01, which is rare in Japanese populations) are associated with the highest susceptibility and radiographic severity, followed by S_3P_ alleles (e.g., *01:01 and *04:05, which are common in the Japanese populations), while S_1_ and S_3D_ alleles may be neutral or even protective [5,6].

Among the various immune cells involved in the immunopathogenesis of RA, monocytes play a central role, particularly through their involvement in inflammation [7]. Human monocytes are a subset of peripheral blood leukocytes characterized by their capacity for phagocytosis, antigen presentation, and the production of proinflammatory cytokines and chemokines, such as tumor necrosis factor-α, interleukin (IL)-1β, IL-6, and C-C motif chemokine ligand 2 [8]. The monocyte-to-lymphocyte ratio (MLR) was previously shown to be significantly higher in patients with RA than in healthy controls [9]. In addition, monocytes exhibit phenotype changes towards a hyper-inflammatory state even at the preclinical stage of RA [10,11]. These findings highlight the pivotal role of monocytes in the early pathogenesis of RA through their proinflammatory activity.

Monocytes have been attracting increasing attention as potential initiators of adaptive autoimmune responses. Based on the surface expression of cluster of differentiation (CD) 14 and CD16, monocytes are classified into three functionally distinct subsets: classical (CD14^++^CD16^-^), intermediate (CD14^++^CD16^+^), and non-classical (CD14^+^CD16^++^) [8]. Intermediate monocytes, which exhibit HLA-DR and potent antigen-presenting abilities, are elevated in patients with RA [12–14]. Thomas et al. recently reported the expression of peptidyl arginine deiminase 4 (PAD4) on the membrane surface of monocytes, and also that monocytes citrullinate their own membrane proteins by their PAD4 and present them as antigens, leading to the production of ACPAs [15]. Based on these findings, monocytes are considered to play a significant role in the pathogenesis of RA by participating in adaptive immune responses.

A genome-wide association study (GWAS) using data from a Dutch non-patient twin-family population revealed a relationship between the HLA-DRB1 allele and monocyte counts [16]. Similar findings were reported by GWAS using data from a Japanese population [17]. Therefore, SE alleles may also have an effect on monocyte counts in patients with RA because they are HLA-DRB1 alleles. However, the relationship between SE alleles and monocyte counts in patients with RA remains unclear.

The present study examined the relationship between SE alleles and monocyte counts in the peripheral blood of disease-modifying antirheumatic drug (DMARD)-naïve patients newly diagnosed with RA. We also investigated whether specific SE alleles were associated with monocyte counts, and how this was related to anti-cyclic citrullinated peptide antibody (anti-CCP Ab) titers. The present results will provide novel insights into the immunogenetic architecture of RA.

## Materials and methods

### Study design and subjects

This was a single-center, retrospective observational study. Inclusion criteria were as follows: patients who fulfilled the 1987 American College of Rheumatology criteria [18], had undergone SE genotyping tests, had available results from laboratory tests at onset, and had never received DMARD. Patients were excluded if they had active infections, malignancies, or hematological diseases at onset. Following the application of these criteria, 86 patients diagnosed with RA at Japan Red Cross Matsuyama Hospital between May 1993 and August 2016 were included in the study.

This retrospective study was conducted in accordance with the Declaration of Helsinki and was approved by the Institutional Review Board of Japan Red Cross Matsuyama Hospital, Japan (approval number: 1157). Given the retrospective observational nature of the study, the need for written informed consent was waived. An opt-out method was used to obtain consent, and information regarding the study was disclosed on the institutional website to provide patients with the opportunity to decline participation. Data collection was conducted between March 21 and March 28, 2025. During data collection, the authors had access to information that could identify individual participants.

### Laboratory tests

White blood cell counts, neutrophil counts, lymphocyte counts, monocyte counts, and C-reactive protein (CRP) levels were obtained from data at the onset of RA. The earliest available measurements for RF titers and anti-CCP Ab titers were used.

All patients were stratified into tertiles based on peripheral blood monocyte counts as follows: low (monocyte count <330.9/μL), intermediate (330.9–479.6/μL), and high (>479.6/μL) monocyte count groups. This classification was used in subsequent analyses to evaluate factors associated with high monocyte counts.

### HLA-DRB1 genotyping and SE classification

HLA-DR genotyping was performed using a polymerase chain reaction/sequence-specific oligonucleotide analysis. The following alleles were defined as positive for SE: HLA-DRB1 *01:01, *01:02, *04:01, *04:04, *04:05, *04:08, and *10:01 [19]. Additionally, we classified HLA-DRB1 alleles as described by Tezenas et al. [5]. Specifically, HLA-DRB1 *01:01, *01:02, *04:04, *04:05, * 04:08, and *10:01 were classified as S_3P_ alleles, whereas *04:01 was classified as the S_2_ allele, with both representing high-risk genotypes according to the SE classification.

### Statistical analysis

Values are expressed as medians and interquartile ranges (IQRs) or as numbers and percentages. Continuous non-parametric values were compared using the Mann-Whitney *U* test between two groups. Categorical values were compared using Fisher’s direct probability test between the groups. A multivariable analysis was performed using a logistic regression model to adjust for confounding factors, including sex and the positivity of specific SE alleles. Steel’s test was applied for multiple comparisons of non-parametric data against a single control group. P values <0.05 were considered to be significant. All analyses other than the multivariate analysis were performed using GraphPad PRISM® Version 9.4.1 (GraphPad Software, San Diego, CA, USA). The multivariate analysis was conducted using JMP software, version 17 (SAS Institute, Cary, NS, USA).

## Results

### Patient characteristics

Patient characteristics are shown in **Table 1**. The present study included 86 Japanese patients with RA. The median age of patients at onset was 56.5 years, and 81.4% of patients were female. There were 62 patients with at least one SE (SE-positive patients), while 24 patients had no SE (SE-negative patients). A detailed assessment of SE-positive patients revealed that 45 were single positive and 17 were double positive. The numbers of SE-positive patients with HLA-DRB1 *01:01, *01:02, *04:01, *04:04, *04:05, * 04:08, and *10:01 alleles were 16, 0, 6, 0, 45, 0, and 2, respectively, while those with the S_2_ and S_3P_ alleles were 6 and 58, respectively.

**Table 1:** Patient characteristics.

Variables	All patients (n = 86)	SE-negative patients (n = 24)	SE-positive patients (n = 62)	P value
Age (years)	56.5 (44.1–67.5)	57.5 (50.8–69.2)	55.8 (43.5–67.3)	0.39
Female, n (%)	70 (81.4)	21 (87.5)	49 (79.0)	0.54
TJC, 0–28	3 (1.5–6)	4 (2–7)	3 (1–5.5)	0.19
SJC, 0–28	3 (1–5)	2.5 (0.3–6)	3 (1–5)	0.71
CRP (mg/dL)	1.32 (0.33–3.62)	1.52 (0.36–3.74)	1.25 (0.32–3.62)	0.81
Anti-CCP Ab positive, n (%)	76 (88.4)	19 (79.2)	57 (92.0)	0.13
Anti-CCP Ab (U/mL)	109.2 (28.5–519.9)	83.4 (9.1–188.7)	163.6 (39.2–712.3)	0.095
RF positive, n (%)	76 (88.4)	21 (87.5)	55 (88.7)	>0.99
RF (IU/mL)	75.0 (29.8–203.5)	63.5 (25.3–206.3)	76.0 (30.0–203.5)	0.87
WBC (/μL)	7645 (5860–9518)	7495 (5040–9038)	7865 (6362–9885)	0.11
Neutrophils (/μL)	5173.4 (3996.8–7050.2)	4904.0 (3135.6–6443.5)	5227.0 (4257.9–7294.8)	0.14
Lymphocytes (/μL)	1440.1 (1134.4–1905.0)	1676.5 (1124.0–2065.4)	1394.7 (1140.0–1905.0)	0.46
Monocytes (/μL)	395.4 (309.4–563.4)	318.0 (294.0–382.3)	438.2 (331.9–606.1)	0.0004
Monocyte fraction (%)	5.7 (4.5–6.7)	5.0 (4.0–6.3)	6.0 (4.8–7.1)	0.027
Monocyte-to-lymphocyte ratio (%)	29.7 (20.4–39.8)	23.3 (15.1–30.1)	33.0 (24.4–43.5)	0.001

Values represent medians (IQRs) or numbers (%).

Continuous non-parametric values were compared using the Mann-Whitney *U* test. Categorical values were compared using Fisher’s direct probability test. P values <0.05 were considered to be significant.

TJC, tender joint count; SJC, swollen joint count; CRP, C-reactive protein; anti-CCP Ab, anti-cyclic citrullinated peptide antibody; RF, rheumatoid factor; WBC, white blood cell; SE, shared epitope.

No significant differences were observed in age or sex distribution between the SE-positive and SE-negative groups. The tender joint count and swollen joint count were similar between the two groups. CRP levels and RF titers were also similar. Anti-CCP Ab positivity was numerically higher in the SE-positive group, and median anti-CCP Ab titers were slightly higher in SE-positive patients. SE-positive patients had significantly higher monocyte counts, larger monocyte fractions, and higher MLR than SE-negative patients. In contrast, no significant differences were observed in total white blood cell counts, neutrophil counts, or lymphocyte counts between the two groups.

### Clinical, serological, and genetic factors associated with monocyte counts

**Fig 1:**
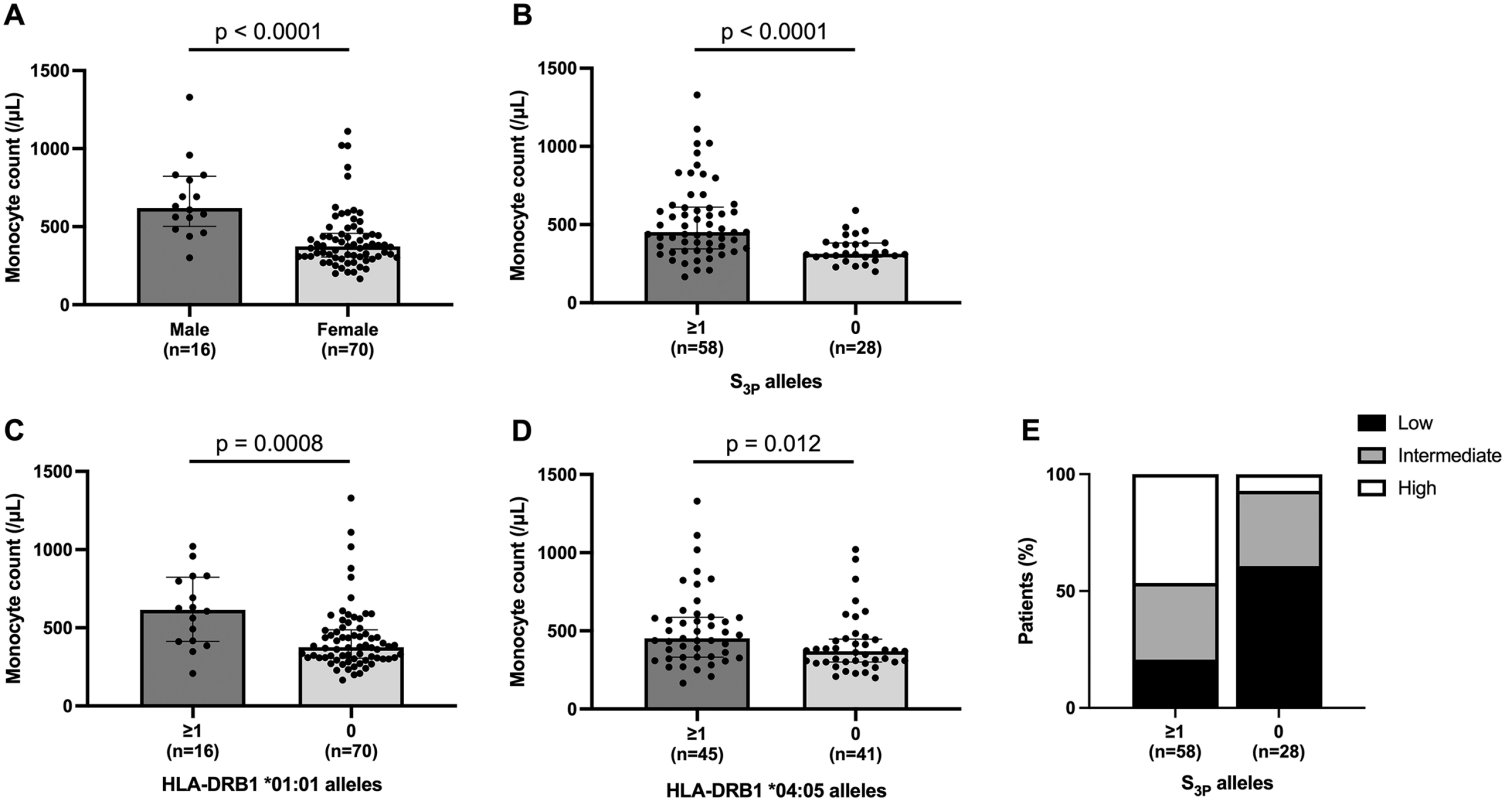
Relationships between monocyte counts and sex and shared epitope alleles. **(A)** Bar graph of monocyte counts between male and female patients. **(B)** Bar graph of monocyte counts between patients carrying at least one S_3P_ allele and patients not carrying S_3P_ alleles. **(C)** Bar graph of monocyte counts between patients carrying at least one HLA-DRB1 *01:01 allele and patients not carrying the *01:01 allele. **(D)** Bar graph of monocyte counts between patients carrying at least one HLA-DRB1 *04:05 allele and patients not carrying the *04:05 allele. **(E)** Distribution of patients with and without S_3P_ alleles across monocyte counts. Patients were classified into three groups based on monocyte counts: low (<330.9/μL), intermediate (330.9-479.6/μL), and high (>479.6/μL). Data represent the median ± IQR. Comparisons were performed using the Mann-Whitney *U* test. P values <0.05 were considered to be significant.

To identify factors associated with peripheral blood monocyte counts other than SE positivity, we compared monocyte counts across clinical and genetic subgroups. Among clinical variables, male patients had significantly higher monocyte counts than female patients (Table 2, Fig 1A). In contrast, no significant associations were observed with age at disease onset, RF or anti-CCP Ab positivity, or CRP levels. Regarding HLA-DRB1 alleles, patients carrying at least one S_3P_ allele showed significantly higher monocyte counts than non-S_3P_ carriers (Table 2, Fig 1B). Similarly, the presence of at least one HLA-DRB1 *04:05 or *01:01 allele was associated with elevated monocyte counts (Table 2, Fig 1C-D, respectively). On the other hand, the carriage of the S_2_ allele (HLA-DRB1 *04:01) was not associated with monocyte levels, and the number of HLA-DRB1 *10:01 carriers was too small for a reliable analysis.

**Table 2:** Factors associated with monocyte counts.

Variables		n (%)	Monocyte count (/μL) (IQRs)	P value
Sex	Female	70 (81.4)	373.4 (305.9–457.2)	<0.0001
	Male	16 (18.6)	619.5 (501.9–822.5)	
Age at onset	<60 years	53 (61.6)	385.2 (305.3–543.2)	0.59
	≥60 years	33 (38.4)	411.8 (312.7–565.1)	
RF	Negative	10 (11.6)	380.5 (291.9–610.9)	0.78
	Positive	76 (88.4)	395.4 (309.6–560.9)	
Anti-CCP Ab	Negative	10 (11.6)	322.4 (285.4–483.8)	0.17
	Positive	76 (88.4)	414.3 (311.2–566.8)	
CRP	Normal	13 (15.1)	347.5 (297.2–455.6)	0.35
	Elevated	73 (84.9)	411.8 (309.6–582.2)	
S_3P_ allele	Patients not carrying S_3P_	28 (32.6)	312.7 (292.1–382.3)	<0.0001
	Patients carrying at least one S_3P_	58 (67.4)	451.6 (344.6–612.0)	
S_2_ allele (HLA-DRB1 *04:01)	Patients not carrying S_2_	80 (93.0)	414.3 (316.8–577.4)	0.096
	Patients carrying at least one S_2_	6 (7.0)	308.5 (286.1–403.5)	
HLA-DRB1 *04:05	Patients not carrying *04:05	41 (47.7)	369.0 (300.6–447.3)	0.012
	Patients carrying at least one *04:05	45 (52.3)	452.0 (331.3–586.4)	
HLA-DRB1 *01:01	Patients not carrying *01:01	70 (81.4)	375.8 (305.9–486.5)	0.0008
	Patients carrying at least one *01:01	16 (18.6)	614.7 (413.2–822.5)	
HLA-DRB1 *10:01	Patients not carrying *10:01	84 (97.7)	395.4 (309.1–566.8)	>0.99
	Patients carrying at least one *10:01	2 (2.3)	406.3 (range: 361.5–451.2) ^†^	

Values represent medians (IQRs) or numbers (%).

Continuous non-parametric values were compared using the Mann-Whitney *U* test. P values <0.05 were considered to be significant.

RF, rheumatoid factor; anti-CCP Ab, anti-cyclic citrullinated peptide antibody; HLA-DRB1, Human Leukocyte Antigen-DR beta 1.

†IQR was not calculated because the number of patients carrying at least one *10:01 was small.

A significant difference was noted in the distribution of the low (<330.9/μL), intermediate (330.9–479.6/μL), and high (>479.6/μL) monocyte categories between S_3P_ carriers and non-S_3P_ carriers (p < 0.0001, **Fig 1E**). The percentage of patients in the intermediate monocyte group was similar in S_3P_ carriers and non-S_3P_ carriers. However, non-S_3P_ carriers were more likely to be classified into the low monocyte group, whereas S_3P_ carriers were more frequently classified into the high monocyte group.

These results suggest that male sex and specific SE alleles, particularly S_3P_ including HLA-DRB1 *04:05 and *01:01, were both associated with increased monocyte counts in patients with RA.

### Multivariate analysis of factors associated with high monocyte counts

To identify independent factors associated with elevated monocyte counts, we performed a multivariate logistic regression analysis using high monocyte counts (>479.6/μL) as the outcome. When sex and the presence of at least one S_3P_ allele were entered into the model, both remained significant: male sex and S_3P_ positivity were independently associated with high monocyte counts (**Table 3, Model 1**). In a separate model evaluating sex together with individual SE alleles, HLA-DRB1 *04:05 and *01:01 both remained significant. The presence of HLA-DRB1 *01:01 showed the highest adjusted odds ratio (AOR), followed by male sex and *04:05 (**Table 3, Model 2**). These results suggest that among the evaluated variables, the HLA-DRB1 *01:01 allele exerted the strongest independent effect on the likelihood of elevated monocyte counts in RA patients.

**Table 3:** Multivariate analysis of factors associated with high monocyte counts.

**Model 1**		
Variables	Adjusted odds ratio (95% CI)	P value
Male	19.5 (3.6–105.4)	0.0006
At least one S_3P_ allele	15.2 (2.5–93.6)	0.003
**Model 2**		
Variables	Adjusted odds ratio (95% CI)	P value
Male	15.2 (2.6–90.1)	0.002
At least one HLA-DRB1 *04:05	14.0 (2.4–82.2)	0.003
At least one HLA-DRB1 *01:01	21.7 (2.9–164.6)	0.002

Values represent the adjusted odds ratio (95% CI).

A multivariable analysis was performed using a logistic regression model to adjust for confounding factors, including sex and the positivity of specific SE alleles. P values <0.05 were considered to be significant.

CI, confidence intervals.

### Relationship between SE alleles and ani-CCP Ab titers

**Fig 2:**
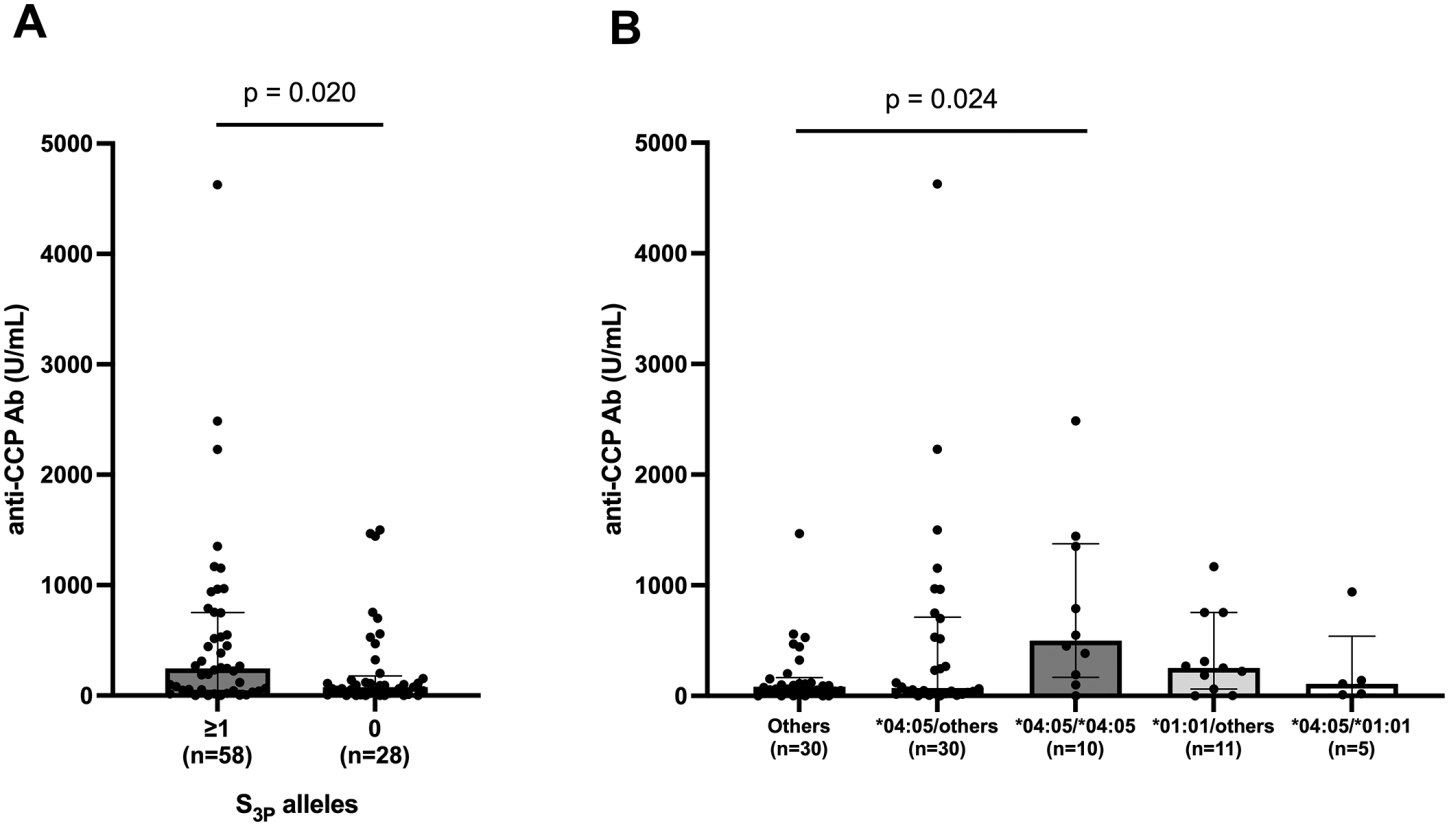
Comparison of anti-cyclic citrullinated peptide antibody (anti-CCP Ab) titers by the positivity of S_3P_ alleles and combination of shared epitope alleles. **(A)** Bar graph of anti-CCP Ab titers between patients carrying at least one S_3P_ allele and patients not carrying S_3P_ alleles. **(B)** Bar graph of anti-CCP Ab titers among five HLA-DRB1 allele combination groups: *04:05/others, *04:05/*04:05, *01:01/others, *04:05/*01:01, and others (patients carrying neither *04:05 nor *01:01). The “others” group was the reference. anti-CCP Ab, anti-cyclic citrullinated peptide antibody. Data represent the median ± IQR. Comparisons were performed using the Mann-Whitney *U* test between two groups. Steel’s test was applied for multiple comparisons of non-parametric data against a single control group. P values <0.05 were considered to be significant.

We examined the relationship between SE alleles and anti-CCP Ab titers in more detail. As shown in **Table 1**, SE-positive patients had slightly higher anti-CCP Ab titers than SE-negative patients. When patients were stratified by the presence or absence of S_3P_ alleles, patients carrying at least one S_3P_ allele had significantly higher anti-CCP Ab titers than non-S_3P_ carriers (median anti-CCP Ab titers, 245.1 U/mL vs. 76.6 U/mL, p = 0.020) (**Fig 2A**). We also compared anti-CCP Ab titers across specific SE allele combinations, including HLA-DRB1 *04:05 heterozygotes, *04:05 homozygotes, *01:01 heterozygotes, the *04:05/*01:01 compound heterozygote, and others (i.e., patients carrying neither *04:05 nor *01:01). Using Steel’s test with the “others” group as a reference, we found that patients homozygous for HLA-DRB1 *04:05 had significantly higher anti-CCP Ab titers (p = 0.024) (**Fig 2B**).

## Discussion

The present study investigated the relationship between SE alleles and monocyte counts at the onset of RA. The main results obtained were as follows: (1) SE-positive patients had significantly higher monocyte counts than SE-negative patients; (2) S_3P_ alleles, particularly HLA-DRB1 *01:01 and *04:05, and male sex were associated with elevated monocyte counts, with HLA-DRB1 *01:01 showing the strongest relationship in the multivariate analysis; and (3) patients carrying at least one S_3P_ allele had significantly higher anti-CCP Ab titers than patients carrying no S_3P_, with a strong relationship being observed in patients homozygous for HLA-DRB1 *04:05.

The present results revealed that SE-positive patients had significantly higher monocyte counts at disease onset than SE-negative patients, which may be mechanistically linked to the genetic regulation of monocyte counts. GWAS studies demonstrated that the HLA-DRB1 region was associated with monocyte counts in both European and Japanese populations [16,17], suggesting a shared genetic basis for monocyte regulation across ethnicities. These findings indicate that monocyte mobilization from the bone marrow to the peripheral blood in SE-positive patients may be enhanced by HLA-DRB1-mediated genetic regulation. In patients with RA, monocyte expansion is skewed towards the intermediate subset (CD14^++^CD16^+^) [12–14], which has a longer lifespan than classical monocytes (CD14^++^CD16^-^), highly expresses HLA-DR, and exhibits a strong antigen-presenting capacity [8,20]. In patients carrying HLA-DRB1 risk alleles, antigen-presenting cells, including monocytes, are considered to present citrullinated antigens more efficiently, leading to the enhanced activation of autoreactive T cells and amplification of adaptive immune responses. This cascade promotes the production of cytokines, such as IL-6, granulocyte-macrophage colony-stimulating factor, and interferon-γ, which stimulate myelopoiesis and enhance the mobilization of monocyte precursors from the bone marrow [10,12,21–23], thereby increasing circulating monocyte counts. Although the precise mechanisms remain unclear, genetic factors within the HLA-DRB1 region may indirectly contribute to monocyte expansion through immune regulatory pathways, particularly in SE-positive RA patients.

In addition to SE positivity, we found that male sex was independently associated with elevated monocyte counts, which is consistent with previous findings on healthy populations [16,24]. Lin et al. reported that among healthy adults, males had higher monocyte counts than females [16]. Starr et al. also demonstrated between the ages of 78 and 87 years, males consistently had higher peripheral monocytes counts than females [24]. These findings suggest that sex is a regulatory factor for monocyte counts. However, in the present study, a multivariate analysis using high monocyte counts as the outcome revealed that the AOR for HLA-DRB1 *01:01 was higher than that for male sex. This result shows that HLA-DRB1, particularly *01:01, was a stronger factor affecting monocyte counts than sex.

Previous studies consistently reported that SE-positive patients exhibited significantly higher anti-CCP Ab titers than SE-negative patients [25,26]. Although this difference was not significant in the present study, possibly due to the limited sample size, SE-positive patients had slightly higher anti-CCP Ab titers than SE-negative patients. Moreover, patients carrying at least one S_3P_ allele had significantly higher anti-CCP Ab titers than non-S_3P_ carriers. When each SE allele within the S_3P_ group was examined, patients homozygous for HLA-DRB1 *04:05 showed a significant increase in anti-CCP Ab titers. In contrast, a significant increase in anti-CCP Ab titers was not observed among patients carrying at least one HLA-DRB1 *01:01, despite its strong relationship with high monocyte counts. Consistent with the present results, previous studies involving a larger number of Japanese patients with RA demonstrated that anti-CCP Ab positivity and titers were significantly higher in patients carrying HLA-DRB1 *04:05, but not in those carrying *01:01 [27,28].

This discrepancy between the effects of HLA-DRB1 *04:05 and *01:01 on anti-CCP Ab titers may be attributed to allele-specific differences in the citrullinome. The RA citrullinome is a group of proteins that have undergone post-translational modifications by PAD, in which arginine residues are converted into citrulline [15]. Among the five PAD isoforms, PAD2 and PAD4 have been identified as the key isoforms responsible for generating citrullinated proteins in RA [29]. Neutrophils are prominent sources of PAD2 and PAD4, and release them into the extracellular space through cell death and neutrophil extracellular trap formation [30]. Other than neutrophils, circulating monocytes also express PAD4, and upon differentiation into macrophages within the synovium, they begin to express PAD2 in addition to PAD4 [31]. Thomas et al. recently demonstrated that enzymatically active PAD4 localized to the plasma membrane of human monocytes [15]. Membrane-associated PAD4 is capable of citrullinating both extracellular proteins, such as fibrinogen, and intrinsic monocyte membrane proteins, including the CD11b/CD18 complex (Mac-1), thereby generating highly immunogenic neoepitopes. This neoepitope in turn leads to the generation of Mac-1-specific ACPAs (Mac-1 ACPAs). Mac-1 ACPAs are distinct from anti-CCP Ab routinely measured in clinical practice. This distinction may explain the different impact of HLA-DRB1 *04:05 and *01:01 on anti-CCP Ab titers even though both alleles belong to the same group, S_3P_. Previous studies suggested that SE alleles affect not only overall susceptibility to ACPA positivity, but also the specificity of the ACPA repertoire. For example, Mac-1 ACPAs were significantly associated with HLA-DRB1 *04:04, whereas anti-CCP Abs were strongly associated with *04:01 [15]. In addition, antibodies to citrullinated fibrinogen were significantly associated with HLA-DRB1 *04:04, whereas*04:01 and *01:01 showed only a trend toward association [32]. These findings indicate that individual SE alleles differentially affect the associated ACPA and also which citrullinome exhibits preferential binding. Therefore, the present results showing that HLA-DRB1 *04:05 and *01:01 exerted distinct effects on anti-CCP Ab titers may be explained by allele-specific differences in their associated citrullinome. The elevated monocyte counts in patients carrying HLA-DRB1 *01:01 may promote the production of antibodies against the monocyte-derived citrullinome, such as Mac-1 ACPAs. Therefore, monocytes may contribute to the early pathogenesis of RA through antigen presentation and the generation of ACPAs; however, the extent of their impact on the composition of the RA citrullinome and subsequent immune responses may vary depending on the presence and type of SE alleles.

The increased number of monocytes may enhance the frequency of antigen presentation, potentially leading to the activation and expansion of T cells in the peripheral blood. However, lymphocyte counts were not elevated, and no significant differences were observed between SE-positive and -negative patients. Du et al. reported that patients with RA had significantly higher peripheral monocyte counts, but significantly lower lymphocyte counts than healthy controls [9]. Another study demonstrated that lymphocyte counts in RA were similar to those in healthy controls [33]. These phenomena may be explained as follows. Not all lymphocyte subsets increase in RA. Large-scale immunophenotyping revealed heterogeneous shifts in peripheral blood [34]. Although the percentage of CD4^+^ effector memory T cells re-expressing CD45RA (TEMRA) was higher than in healthy controls, the percentages of several other T-cell subsets, such as CD4^+^ central memory T cells, CD8^+^ naïve T cells, and CD8^+^ effector memory T cells, were lower. Furthermore, activated T cells migrate from the bloodstream into inflamed synovial tissue through interactions with endothelial cells at postcapillary venules in RA [35]. Consequently, T cells become enriched in the inflamed synovium rather than accumulating in the circulation [36]. Collectively, these findings provide support for peripheral lymphocyte counts not necessarily increasing even if monocyte-mediated antigen presentation is enhanced in SE-positive patients.

This study has several limitations that need to be addressed. This was a single-center, retrospective study conducted exclusively on a Japanese population, which may limit the generalizability of the results obtained to other ethnic groups. Furthermore, it was not possible to adequately evaluate SE alleles that are less prevalent in the Japanese population, such as HLA-DRB1 *10:01. However, the regulation of monocyte counts by HLA-DRB1 has been demonstrated in both European and Japanese populations, supporting the broader relevance of this relationship. Another limitation is the small sample size. Moreover, due to the retrospective design, monocyte subsets were not analyzed because peripheral blood mononuclear cells at disease onset were not preserved. In addition, Mac-1 ACPAs were not measured and, thus, their potential contribution remains unclear. Finally, we did not investigate SE alleles or monocyte counts in healthy Japanese controls in the present study. However, data from large-scale studies on Japanese populations are available. Ikeda et al. analyzed 18,604 healthy individuals and reported that approximately 21–22% carried SE alleles [37]. Takami et al. examined 750 healthy Japanese adults and showed that the median absolute monocyte count was approximately 300/μL [38]. Although a direct statistical comparison was not feasible, the frequency of SE alleles and monocyte counts were both higher in RA patients, particularly in SE-positive RA patients, in the present study than in healthy Japanese controls. These results further support SE alleles affecting peripheral blood monocyte counts. However, a multinational, multi-center, prospective study is needed in the future to address all these limitations.

In conclusion, this is the first study to demonstrate that SE alleles are associated with peripheral blood monocyte counts at disease onset. The present results suggest the involvement of monocytes in the early pathogenesis of RA, particularly in patients carrying S_3P_ alleles. This study provides novel insights into the immunogenetic mechanisms underlying the pathogenesis of RA.

## Supporting information

S1 File All data. (XLSX)
